# Cerebral microbleeds (CMBs) – relevance for mechanisms of cerebral hemorrhage – analysis of 24 MRI evaluated patients

**Published:** 2013-12-25

**Authors:** D Ghelmez, S Sorin Tuţă, C Popa

**Affiliations:** * National Institute of Neurology and Neurovascular Diseases, Bucharest; ** ”Carol Davila” University of Medicine and Pharmacy, Bucharest

**Keywords:** cerebral microbleeds, CMBs, small vessel disease, intracerebral hemorrhage

## Abstract

Abstract

Introduction. The new MRI techniques introduced in the last decade allowed the detection of cerebral microbleeds (CMBs) in different groups of diseases: stroke, Alzheimer disease, vascular dementia or healthy people of advanced age. CMBs are radiologically defined as small, rounded, homogeneous, hypointense lesions on T2*-weighed gradient-recalled echo (T2*-GRE) sequences.

Objective and Method. We evaluated the prevalence, number and location of CBMs in a cohort of 26 consecutive cerebral hemorrhage patients admitted in the National Institute of Neurology and Neurovascular Diseases. We also assessed the association between CMB, classical vascular risk factors and small vessel disease.

Results and Conclusions. From the 26 patients, 2 patients had secondary intracerebral hemorrhage (ICH) (hemorrhage in metastasis, respectively a cavernoma). From the 24 ICH patients 12 have had at least 1 CMB lesion. The average volume of the cerebral hemorrhage was larger in patients with CMBs, with a relative increase of 42%. Small vessel disease was associated with a significant increase in the presence of CMBs (relative increase of 86%). In both cases, however, since the number of patients enrolled was small, the correlations did not reach statistical significance.

## Introduction

The new MRI techniques introduced in the last decade allowed the detection of cerebral microbleeds (CMBs) in different groups of diseases: stroke, Alzheimer disease, vascular dementia or healthy people of advanced age. CMBs are radiologically defined as small, rounded, homogeneous, hypointense lesions on T2*-weighed gradient-recalled echo (T2*-GRE) sequences [**1**]. SWI technique, recently introduced into practice is a high resolution, three-dimensional T2 *-GRE with a very high sensitivity for CMBs (and bleeding in general). Another benefit of the SWI sequence is that it can differentiate CMBs from calcification or subarachnoid space vessels, all appearing as hypointense lesions on T2 *-GRE [**2**,**3**]. The histopathological examination showed that CMBs are small hemorrhages adjacent to small caliber abnormal vessels affected by cerebral amyloid angiopathy (CAA) or hypertensive angiopathy [**4**]. 

## Objective and Method

We evaluated the prevalence, number and location of CBMs in a cohort of 26 consecutive cerebral hemorrhage patients admitted in the National Institute of Neurology and Neurovascular Diseases. We also assessed the association between CMBs, classical vascular risk factors and small vessel disease. Besides the emergency cerebral CT scan, brain MRI was performed in these patients, including beside the classic sequences, the special sequences of SWI or T2 *-GRE. According to the data found in literature, CMBs are associated with both ischemic stroke, especially lacunar stroke, and with cerebral hemorrhage, hypertensive or the CAA type [**5**].

## Results

From the 26 patients with ICH, 12 have had at least 1 CMB lesion: 1 patient had one lesion, 4 patients two CMBs, 1 patient had 3 CMBs and the rest of the patients more than 3. 26 patients were included in the study but 2 of them proved to have secondary hemorrhages (hemorrhage in metastasis and, respectively, a cavernoma). All 12 CMB patients were hypertensive and 9 of them had other signs of small vessel disease (lacunar strokes and/or leukoaraiosis). Regarding the ICH topography there were two cerebellar hemorrhages, one brainstem hemorrhage, three lobar hemorrhages, 8 hemorrhages in the basal ganglia and 10 thalamic hemorrhages. 

**Fig. 1:**
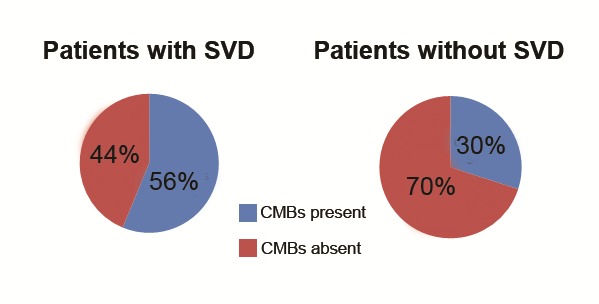
Prevalence of CMBs in patients with small vessels disease

We analyzed the prevalence of CMBs in patients with cerebral small vessels disease (SVD) (**Fig. 1**). Within our group, small vessel disease (lacunar strokes and/or leukoaraiosis) was associated with a significant increase in the presence of CMBs (relative increase of 86%). Given the small number of patients in the group, we tried to use the exact Fischer test [**6**] to assess the statistical significance of the observed association. The P-value obtained was 0.24, so, although the presence of small vessel disease appears to be associated with a higher prevalence of CMBS, our data did not reach statistical significance. 

**Fig. 2:**
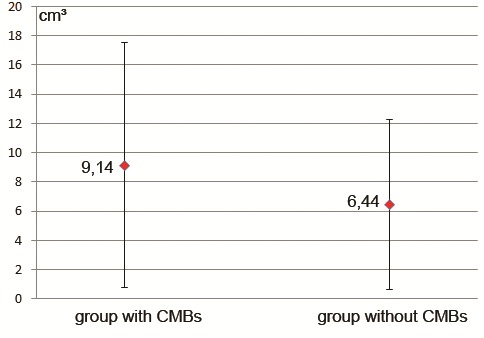
ICH volume (cm₃) in patients with and without CMBs. The large superposition of the variance standard deviations is obvious

The average volume of bleeding was higher in patients with CMBS, 9.1 cm₃vs. 6.44 cm₃ (relative increase of 42%). To compare the means we performed a unifactorial analysis of variance (ANOVA) and contrary to our expectations the mean variance was relatively homogeneous (F1, 22 = 0.84) (**Fig. 2**), but this result, again, failed to reach statistical significance (P value 0.37).

**Fig. 3:**
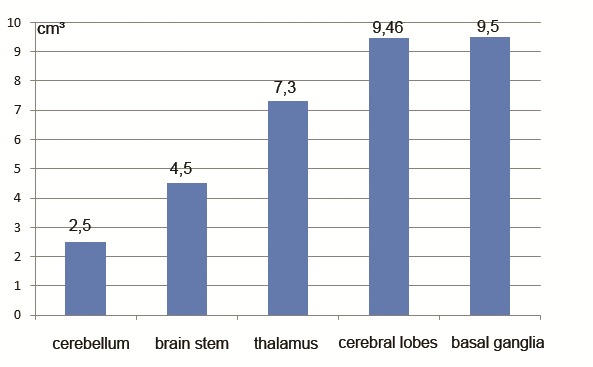
Average volume of ICH (cm₃) according to location

**Fig. 4:**
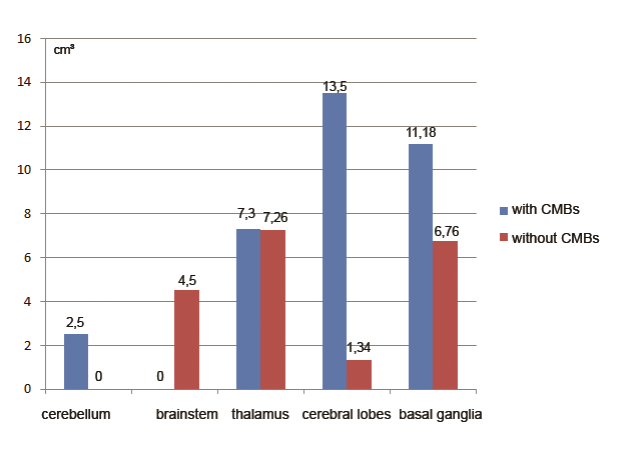
The average volume of ICH (cm₃) according to location and presence of CMBs

We tried to analyze the differences between the average volume per location depending on the presence or absence of CMBs (Fig. 3,4). There is a tendency for the growth of the volume in the locations that had a greater number of cases, but the rare locations made the association not to reach statistical significance. 

**Table 1:**
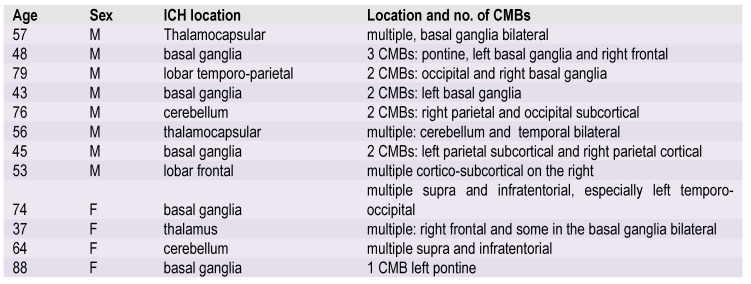
Topography and number of CMBs

## Discussion

Although in this case there was only a tendency in the increase of the volume of hematoma in patients with CMBs but without reaching statistical significance, this trend was confirmed by other studies. According to one study, in patients with lobar or basal ganglia hemorrhages, the lesional volume was two to three times larger in patients who associated CMBs than in the control group [**7**]. This fact is important in clinical practice to quantify the risk of secondary ICH in patients receiving antithrombotic therapy. The presence of CMBs is an independent risk factor for secondary ICH in patients with oral anticoagulation or those undergoing thrombolytic therapy. A study that enrolled patients with oral anticoagulation, an arm with secondary ICH and the other arm without ICH, showed that a higher percentage of patients had CMBs lesions on MRI imaging in ICH group compared to the control group and that the number of CMBs was also higher in the first case [**8**]. With regard to the thrombolytic treatment, the results of a BRASIL study showed a 5.8% brain hemorrhage in patients with CMBs compared with 2.7% in those without CMBs [**9**]. Another multicentered study tried to stratificate the post-thrombolysis risk of cerebral hemorrhage in patients with acute ischemic stroke, who associated CMBS lesions on MRI. There was a trend towards increasing the risk of cerebral hemorrhage with a borderline p-value (p = 0.05) but the bleeding risk had significantly increased with the increase in the number of CMBs. In the subgroup of patients with a number of more than 10 CMBs, the cerebral hemorrhage rate was 29% [**10**].

## Conclusion

Of the 24 patients with primary ICH, half of them had at least one CMB lesion. All the patients with CMBs were hypertensive. The average volume of the ICH lesions was larger in patients with CMBs with a relative increase of 42%. Small vessel disease was associated with a significant increase in the presence of CMBs (relative increase of 86%). However, in both cases, since the number of patients enrolled was small, the correlations did not reach a statistical significance. CMBs presence, detected by modern imaging techniques (MRI-SWI), appeared to be an important factor in assessing the risk of hemorrhagic stroke in patients with anticoagulant or thrombolytic therapy for ischemic stroke. 
